# Widening interest, widening participation: factors influencing school students’ aspirations to study medicine

**DOI:** 10.1186/s12909-018-1221-3

**Published:** 2018-05-30

**Authors:** Alexander J. Martin, Benjamin J. Beska, Greta Wood, Nicola Wyatt, Anthony Codd, Gillian Vance, Bryan Burford

**Affiliations:** 1 Newcastle University, Newcastle-upon-Tyne, UK; 2 School of Medical Education, Newcastle University, Ridley Building 1, Newcastle-upon-Tyne, NE1 7RU UK

**Keywords:** Widening participation, Selection, Medical careers, Widening access, Medical school admissions

## Abstract

**Background:**

Under-representation of some socio-economic groups in medicine is rooted in under-representation of those groups in applications to medical school. This study aimed to explore what may deter school-age children from applying to study medicine.

**Methods:**

Workshops were undertaken with school students aged 16–17 years (‘Year 12’, *n* = 122 across three workshops) and 13–14 years (‘Year 9’, *n* = 295 across three workshops). Workshops used a variety of methods to identify and discuss participants’ perceptions of medicine, medical school and the application process. Year 12 workshops focused on applications and medical school, while Year 9 took a broader approach reflecting their relative distance from applying. Subsequent workshops were informed by the findings of earlier ones.

**Results:**

The main finding was that potential applicants had limited knowledge about medicine and medical school in several areas. Older students would benefit from accessible information about medical degrees and application processes, access to work experience opportunities and personal contact with medical students and junior doctors, particularly those from a similar background. Younger students demonstrated a lack of awareness of the breadth of medical careers and a limited understanding of what medicine encompasses. Many Year 9 students were attracted by elements of practice which they did not associate with medicine, such as ‘talking to people with mental health problems’. An exercise addressing this elicited an increase in their interest in medicine.

These issues were identified by participants as being more marked for those without knowledgeable support at home or school. It was apparent that school teachers may not be equipped to fill these knowledge gaps.

**Conclusion:**

Gaps in knowledge and support may reflect the importance of ‘social capital’ in facilitating access to medical school. Medical schools could act as hubs to introduce students to resources which are essential for widening participation. Outreach and support to schools may ensure that fundamental knowledge gaps are equitably addressed for all prospective applicants.

More generally, a focus on medicine which under-emphasises aspects of medical practice involving communication may deter some students and have longer term impact on recruitment to careers including general practice and psychiatry.

## Background

The under-representation of students applying for and studying medicine from less advantaged socioeconomic backgrounds is an ongoing area of concern. Across the countries of the UK, approximately 75% of applicants to medicine have a parent in the highest occupational socio-economic group, and 19.7–34.5% applicants live in the most affluent postcode decile versus 1.8–5.7% in the least affluent [1]. The issue has been raised by governmental reports into social mobility [2, 3] and the Medical Schools Council, the corporate body for medical schools, is examining how recruitment processes may address this imbalance following its ‘Selecting for Excellence’ report [4]. This is not only a concern in the UK, but has been described and prioritised internationally [5–9].

‘Widening Participation’ (WP) is used to describe both the principle of increasing engagement in medical education and the schools and students who are targeted through related initiatives. Encouraging and facilitating applications from all sections of society may benefit the workforce by presenting a wider pool of applicants without the need to reduce standards [10]. Doctors recruited from lower socio-economic status (SES) backgrounds are more likely to work in deprived areas [11] and pursue under-supplied careers such as general practice [12].

Medicine is typically regarded as a ‘selecting’ rather than ‘recruiting’ subject, with more applicants than places. However, under-represented groups are also under-represented in applications. Of those taking the United Kingdom Clinical Aptitude Test (UKCAT), required by most UK medical schools for school-leaver entry, 80% come from higher socioeconomic groups [1]. Students from fee-paying private schools are more likely to be accepted into medicine independent of academic achievement [13]. Medicine, dentistry and veterinary science are the university subjects with the lowest proportion of disadvantaged applicants, with 4.3% applicants from a disadvantaged background compared to an average of 10.9% for other subjects [3]. It appears that structural inequalities exist *before* engagement with the admissions process.

Such an imbalance in applications cannot therefore be addressed purely through selection processes and the consideration of students’ backgrounds during selection [14]. Earlier intervention to improve the equity of applications is necessary, as evidence suggests SES impacts education from an early age [15] and by 14–16 years old academically able, disadvantaged students see medical school as ‘culturally alien’ [16]. Medical schools should therefore explore what can be done to encourage able students from all backgrounds to consider medical careers.

Literature has considered how to encourage wider applications, for example information outreach [17]. However, some admissions staff have concerns about the impact of Widening Participation on medical school reputation and view it negatively, ranging from a box-ticking exercise to undesirable social engineering [18]. Schools also neglect to evaluate the impact of Widening Participation activities [18]. This may in part explain why there has been limited impact reported to date [19].

This project aimed to identify barriers or deterrents to applying for medical school perceived by prospective medical students and explore how those barriers may be addressed, at what stage in school careers, and by which agency.

### Research questions

The project was designed as exploratory research to consider the broad research questions ‘What barriers to application to medical school are identified by school students?’ and ‘What actions, and by which agencies, may mitigate those barriers?’. The focus is on understanding students’ perspectives, rather than evaluating existing interventions.

### Context of the study

This project was embedded within the timetable of an existing programme of Widening Participation activity delivered by the Faculty of Medical Sciences at Newcastle University – Medicine and Dentistry (MaD) days – to access participants. However, the research was developed and conducted independently of the organisers of the MaD days.

MaD days, which are held in Newcastle University Medical School, offer didactic and practical sessions for school students in Year 9 (age 13–14) and Year 12 (age 16–17). Three days per year group take place each academic year. Those attending Year 12 MaD days have usually expressed interest in medicine, whilst those in Year 9 have been nominated by their teachers as potentially having the aptitude to study medicine or dentistry. The Year 12 days are therefore wholly focused on medicine (with separate days for dentistry). The Year 9 days have a morning or afternoon on medicine and the other half of the day on dentistry. This research focused only on medicine.

Recruitment of Widening Participation schools and students is prioritised for MaD days; however, school engagement varies, meaning representation of particular socioeconomic profiles cannot be assured. Five criteria for Widening Participation status are used: home postcode, studying at a school which is part of an established Widening Participation programme, receipt of free school meals, being in local authority care and whether either parent has completed higher education. These criteria reflect the types of contextual data – community, school and individual – identified in the Selecting for Excellence report [4].

## Methods

### Pilot study

In order to develop and sense-check materials for the first of the Year 12 sessions, a pilot session was carried out with current medical students (*n* = 12) and junior doctors (*n* = 2). This was conducted by authors BB and AC, before the appointment of other authors as student interns. Some pilot session participants self-identified as being from Widening Participation backgrounds or schools, but their Widening Participation status was not formally recorded.

Using a method based on the nominal group technique, with questions answered individually on paper and then discussed with the group, participants were asked to consider what had attracted them to medicine, and what could have deterred them. They were asked to focus on recall of their own experience. Responses were used to inform the materials used in the research sessions.

### Research sessions

Research sessions were carried out on each of the six MaD days – three with Year 9, three with Year 12. The project methodology was necessarily pragmatic to fit within the existing MaD day timetable. Principles of action research were used, with the outputs of each research session being fed back into subsequent iterations [20].

### Year 12 sessions

Year 12 MaD days took place in November–December 2015. Each research session was limited to approximately 40 min and took place following the MaD day activities. Session 1 addressed the attractions and deterrents to medicine and identified potential solutions. This fed into Session 2 which further elaborated solutions and their sources and provided the focus for Session 3.

#### Session 1

Session 1 was based around a card-sorting task to identify and prioritise deterrents to choosing medicine. The deterrents identified in the pilot session were used as prompts to simplify the task and reduce pressure on participants, but participants were encouraged to provide commentary on those prompts and generate new deterrents.

A pre-session questionnaire contained free text questions about attractions and deterrents to studying medicine. The primary goal of this was to prime participants to focus on the topic, but the questionnaires were quickly reviewed by researchers before the card sorting tasks in case any substantially different factors had been identified. Questionnaires also recorded participants’ names to link to Widening Participation data collected during MaD day registration.

Participants were divided into four facilitated groups of nine. Each group sorted the deterrents along a scale of importance, discussed decisions as a group and removed cards felt to be irrelevant. Participants could add further deterrents to the set.

#### Session 2

Session 2 aimed to understand the reasoning behind Session 1 deterrents, elaborate how they may be addressed and where responsibility for those solutions may lie – with the individual participant, the school, medical school or elsewhere.

A pre-session priming questionnaire was again used to focus participants and to record their names. Data from Session 1 were used to produce eight specific deterrent statements, which were pinned to a board and used as the basis of discussion (Table 1). In group discussions participants were asked whether they agreed with the deterrents listed. After 10 min, cards were added to the board to prompt suggestions of the agencies which could deliver solutions. Audio-recording supplemented field notes and the pre-session questionnaire.

**Table 1:** Mapped Session 2 prompts

Prompt	Category based upon
I don’t know enough the application process	Application process
It’s competitive: I don’t want to waste a UCAS choice	Application process
I don’t know how to get work experience	Application process
I think there are problems in the NHS and the future is uncertain	Careers
I might find the course too difficult	Course content
I don’t know enough about the course: I may change my mind	Course content
I don’t know how I will pay for the course	Finance
I may not fit in	Social

#### Session 3

Session 3 aimed to elaborate areas which the medical school has greatest capacity to influence, rather than relying on the influence of schools, NHS Trusts or national policy. It consisted of five concurrent focus groups with 6–7 participants addressing three issues raised in Session 2 that were identified as within the purview of the university: the application process, work experience, and course content. Groups discussed each question for around 15–20 min.

### Year 9 sessions

Year 9 sessions aimed to understand perceptions of medicine held by younger, academically able students and the factors influencing their aspirations. They were integrated into the half-day MaD activity as one of four 20-min stations, meaning that time was more limited than in the Year 12 MaD days. Sessions were repeated four times on each day with around 25 participants in each (meaning around 100 participants on each day). Schoolteachers were present in and contributed to discussions. No pre-session questionnaires were used with Year 9 participants.

The findings from Session 1 directly informed the revision of the approach used in both Sessions 2 and 3. Interactive group tasks were used to facilitate discussion.

#### Session 1

The first session sought to establish whether participants were considering medicine as a career and their reasons for this. Participants were asked ‘Would you like to be a doctor?’, ‘Can you see yourself as a doctor?’ and ‘Can you see yourself as a medical student?’ and asked to sort themselves along a physical ‘scale’ by standing beside the response card which best described their feeling: ‘Definitely not’, ‘Probably not’, ‘Maybe’ and ‘Definitely’. This placement was used to prompt discussion and not recorded. Students anonymously wrote down their reasons for their initial position and if their response differed between questions they were asked to explain why. Groups were facilitated by pairs of medical student researchers (AM, GW, BJB, NW), with the other pair and BB observing and taking notes.

#### Session 2 and 3

Session 2 elaborated the identification of limited knowledge among participants and investigated whether a short exercise could change views and aspirations. This format proved useful and was retained for Session 3.

The session began with the same ‘Do you want to be a doctor?’ question as Session 1. This time the number of participants at each ‘scale’ point was recorded. Two new activities then established students’ understanding of medical roles. In the first, a set of cards printed with nine diverse medical job titles (eg ‘paediatrician’, ‘forensic pathologist’) and nine non-medical titles (eg ‘nurse’, ‘osteopath’) were distributed among the group, who were asked to place them on a whiteboard under headings ‘Doctor’ and ‘Not a Doctor’. These were then reviewed as a group, followed by a debrief where researchers explained each job.

The second activity focused on sorting 33 activities selected to reflect a range of medical specialties (eg ‘Prescribing medicines to people’, ‘Organising the delivery of healthcare in a region’) into ‘Doctor’ and ‘Not a doctor’ headings. Once participants as a group had agreed the distribution of cards across these categories, debriefing explained that every activity was potentially part of medicine.

Participants were then asked to indicate activities that most appealed to them by fixing adhesive dots to the cards. They could add two dots to a single activity, select two activities, or use one or neither of the dots.

Finally, the question from the beginning of the session was repeated, and participants again asked to sort themselves along the ‘Definitely not’ – ‘Definitely’ scale and the number at each position recorded.

Data were recorded through field notes, photographs of sorted cards, and the cards complete with adhesive dots which were returned to the researchers.

### Data analysis

Analysis was adapted to the data collected in each session. Qualitative data, captured through written responses, audio recordings and field notes, were analysed by content analysis – identifying and applying summary codes to statements. Data were summarised by BB, with coding, interpretation and application to subsequent sessions discussed and agreed by all authors by email and in face-to-face meetings between sessions. Illustrative quotes are given in the results where appropriate.

Quantitative data were recorded as the ordinal placement of items in sorting tasks, and frequencies of responses in group activities.

### Ethical approval

This study was approved by the Newcastle University Faculty of Medical Sciences Research Ethics Committee (Reference: 00906/2015). All participants and parents were sent an information sheet and consent form for participation and audio recording. Year 12 students, being over 16 years old, could provide written assent without parental agreement. Students for whom consent or assent was not provided did not take part in the research sessions.

## Results

### Year 12 sessions: Results

#### Participants

One hundred and twenty two of 164 (74%) attendees at the three MaD days participated in the research. 84 (74%) participants who provided demographic data were female and 29 were male. The sexes of those who attended but did not take part in the research are not known, but of those registered for the MaD day (not all of whom attended), 72% were female (*n* = 138). Those who took part met slightly more Widening Participation criteria than those who were registered but did not take part (a mean of 1.6 compared to 1.4).

Table 2 summarises the participants with the number of Widening Participation criteria reported to the Faculty of Medical Sciences. Of the 113 (92.6%) participants for whom Widening Participation data were available, 95% met at least one of the five Widening Participation criteria and 46% met more than one. A challenge of conducting studies such as this during an outreach programme is demonstrated by the fact that just 10% of participants in Session 3 had > 1 Widening Participation criteria, meaning the results of this session need to be treated with caution.

**Table 2:** Numbers of participants and those meeting Widening Participation criteria at each Year 12 session

	Number attending MaD day	Number taking part in research (%) ^a^	Frequency (and % of session sample) of each of five Widening Participation criteria among respondents					n with Widening Participation > 1 (% of sample)
			Postcode	School	Free School Meals	Care	Parents	
Session 1	44	36 (82%)	25 (69%)	17 (47%)	10 (28%)	1 (3%)	26 (72%)	28 (77%)
Session 2	60	46 (77%)	29 (63%)	7 (15%)	4 (9%)	0	23 (50%)	24 (52%)
Session 3	60	40 (67%)	15 (38%)	7 (18%)	3 (8%)	0	19 (48%)	4 (10%)
Total	164	122 (74%)	69 (57%)	31 (25%)	17 (14%)	1 (1%)	68 (56%)	56 (46%)

^a^ Including participants who did not provide details to link to Widening Participation indices (Session 1 = 3 students, Session 2 = 6 students)

#### Session 1

Deterrents were coded into eight categories reflecting distinct areas of concern, supplemented by free text comments on the questionnaire (see Table 3). Field notes provided context and clarification on how deterrents were interpreted. Coding was deliberately broad to simplify the deterrents into categories that would be meaningful to subsequent participants. Most focused on process and context.

**Table 3:** High priority deterrents identified in Year 12 Session 1

Deterrent (number of groups ranking deterrent in top five)	Category of deterrent
I don’t think I’ll get in (4)	Anticipation of application process
I might not get the grades (4)	Anticipation of application process
I think there are problems in the NHS (3)	Political context
Medical students are from a different background to me and I won’t fit in (3)	Social background
Having to do the UKCAT (2)	Anticipation of application process
Studying medicine is expensive (2)	Financial cost
Five years is a long course (1)	Concerns about course
Having to have an interview to get in (1)	Anticipation of application process
I don’t know if I could be a doctor (1)	Social background
I don’t know how I will pay for the course (1)	Financial cost
I may not like the subject (1)	Concerns about course
I might find the course difficult (1)	Concerns about course
My family don’t know how to support me in applying (1)	Social background
My school don’t have anyone who can give me advice about applying (1)	Social background
Negative stories about doctors or medicine in the news (1)	Political context
I don’t know how to get work experience (1)	Anticipation of application process

The number in brackets indicates the number of groups (out of 4) that ranked the deterrent as among their ‘top five’, meaning that those at the top indicate more consensus

Deterrents coded as ‘anticipation of the application process’ related to challenges which would arise before entering medical school. Some ostensibly reflected students’ perceptions of their own ability (for example in relation to required grades), but these were generally discussed in terms of the competitiveness of the process and whether an application to medicine represented a ‘good use’ of limited application choices, so focused on process rather than aptitude.

Deterrents relating to ‘concerns about the course’ included uncertainty and apprehension about the course difficulty and duration. Concerns around ‘financial cost’ – course fees and living costs across a course longer than most degrees – also arose. Those arising from ‘political context’ included perceptions of medical careers arising from a bigger picture, including media representations and the societal image of medicine.

Other categories were less clearly linked to time, in that they could have relevance in the short and longer term. ‘Social background’ deterrents centred on comparison with perceived stereotypes and concerns about fitting in at medical school and disparity between the students’ social background and the social and class status they felt doctors represent. References to knowledge gaps arising from a lack of knowledgeable support from school and family were common across all codes.

The ranking of each deterrent’s priority was noted, and those which were rated as being within the top five priority deterrents by any of the groups are summarised in Table 3. Those that were not agreed as priorities by groups were still relevant to individual participants. These included negative experiences of hospitals, lack of encouragement to apply for medicine and concerns about coping with unwell people.

Additional deterrents added by respondents included the availability and attraction of other careers and perceived over-emphasis on academic qualities over personal ones. There was also reference to teachers doubting that students would get the necessary A-level grades for admission, meaning that it would not be worthwhile applying. It may be that such predictions are accurate, but the fact it is a substantive barrier indicates that predictions can have material impact on student decisions.

Session 1 confirmed that the deterrents identified by current medical students in pilot work were relevant to Year 12 students.

#### Session 2 and 3

Free text responses to the questionnaire and the annotated boards in Session 2 were reviewed to identify common solutions. These represented the eight areas brought forward from Session 1 with emphases on the application process, work experience, knowledge of the course, finance, and long-term prospects. Session 3 focused on the application process, work experience and course content.

#### Application process and work experience

While Widening Participation status could not be linked to individual responses, many students associated others’ social background with advantage in terms of school support and knowledge in the application process.

*“We don’t have careers advice so we’ve got to do everything ourselves. Teachers do help but they don’t know about everything”.*

*“People who go to private schools will be schooled in how to answer questions at interviews”.*

This perception was linked to their reported confidence in engaging with the process, and it seemed some felt that the challenges were overwhelming.

*“None of my family have ever been to [university] … and my sixth form is really small…so I wouldn’t know where to start”.*

Student awareness of pre-application aptitude tests was poor, with uncertainty about what the tests involved, cost (indeed, that there are fees and a bursary system), that they are time-limited and that there are rules on resitting. For some, the MaD day was the first time they had heard of the UKCAT.

Some students expressed uncertainty about how to prepare an application and what to include. They identified a need to ‘stand out’, indicating awareness of the competitiveness of the process.

Websites were the primary route to information on the application process identified in Session 3. Some participants took specific, targeted approaches such as searching directly for a medical school or navigating from the university front page. Others started more broadly, such as the national university applications website. None were aware of other national online resources such as those on the Medical Schools Council website. Just two participants mentioned the medical school’s social media presence. Students felt that outreach events would benefit from a take-home, hard-copy resource providing key facts.

Students felt that work experience would be valuable for them but were unclear about the role it plays in admissions decisions and how to access it. Few in both Session 2 and 3 had explored it in detail. This was felt to be a specific area of inequality, with some students able to arrange work experience through family and friends and some schools providing support. There were also barriers in the way Trusts offer and arrange work experience.

*“My local trust doesn’t really offer work experience, but [other trust] does, but they preserve it for people in the area. It would be helpful if they worked together”.*

Students’ age was a perceived barrier to being trusted in a clinical environment by staff who did not know the students; hospital and general practice (GP) policies echo this concern [2].

Respondents had a limited view of relevant work experience, with few identifying that volunteering in non-medical settings could be relevant experience. Many identified ‘work experience’ exclusively with a specific, timetabled period arranged by their school and had not considered gaining experience outside that.

#### Course content

Participants felt they did not have a good understanding of what the course involves. Uncertainties about the academic challenges of Higher Education and the change in learning style were common.

*“You’re used to having a teacher there all the time so it’s different when you come to [university], they’re not going to be there”.*

There were also questions about student life and adjustment to university, e.g. finding accommodation. This social aspect was another area where a lack of access to first-hand knowledge from parents, siblings or friends may add to uncertainty.

The opportunities provided by MaD days to speak to medical students were valued, compared to open days held by more senior staff. First-hand accounts, especially from those with similar backgrounds, would be helpful.

#### Finance

Students expressed uncertainty about student loans, fees, repayments and the availability of support. They expressed interest in information on financial management over a long university course, and again felt that first-hand accounts from current or recent medical students would provide valuable insight.

#### Careers

While many of the concerns surrounded applying to medical school, longer term views about medicine as a career were also relevant. Medicine was perceived as different to other courses and careers.

*“I think it should be shown more as a vocation rather than just any old career path, it requires more than just turning up”.*

Topics of work-life balance and stress were raised alongside recognition of the intrinsic rewards of working in medicine. There was, surprisingly, a perception of medicine as a ‘narrow’ career with few options – something that was explored further in the Year 9 sessions. Participants also demonstrated a degree of political awareness about current issues within the NHS which translated into concerns about longer-term prospects.

#### Participant-proposed solutions

Lack of information was the root of most deterrents. There was a strong feeling that hearing from first and second year medical students would best help understanding the application process, while older students, junior doctors and faculty could best explain the course and career options.

The idea of peer support for applicants was raised. Some participants lacked school peers applying for medicine and stated that informal peer contacts made on the MaD day were a useful source of support. This sense of peer community could be enabled by more visits to the medical school or a virtual community or forum for potential applicants. Third-party online forums for applicants do exist, but these were not mentioned by students.

Some students felt that the pressures of A-level study limited their time for attending events and that outreach visits to their school may be more time-efficient. Students felt sessions could provide opportunities to practice interviews or multiple mini interviews.

Several participants identified a potential role for the medical school in arranging work experience by acting as an intermediary in identifying and facilitating access to work experience placements.

### Summary of year 12 sessions

The Year 12 sessions identified and elaborated information needs among prospective applicants. These included short-term considerations such as gaining work experience, details of the application process and finance, through information about course content and being a student, to longer term questions about medical careers. These issues were exacerbated for participants who felt their schools were isolating for medicine applicants, and staff less knowledgeable and supportive.

### Year 9 sessions: Results

Year 9 sessions were repeated four times on each day with around 25 participants in each (meaning around 100 participants on each day). No personal details were obtained from these participants. The approximate gender distribution was 58% female (*n* = 170). Full Widening Participation criteria for these participants were not available, but of free school meals, being in care, or having parents who did not complete higher education, 134 met one criterion, 22 met two, and 2 met all three.

### Session 1

Participants’ positions changed between the three questions (‘Would you like to be a doctor?’, ‘Can you see yourself as a doctor?’ and ‘Can you see yourself as a medical student?’), indicating a difference in interest, perceived capability, and understanding of medical careers. We cannot rule out that some moved because they felt they were expected to, but most could articulate reasons for changing their position, suggesting credibility of responses.

Some in the ‘Definitely not’ or ‘Probably not’ groups were simply uninterested in medicine. However, others referred to specific deterrents such as squeamishness around blood and ‘gore’, and not wanting to have the ‘life and death’ responsibility involved in medicine. These responses indicate how medicine is perceived at this age, with a partial and limited view of what medicine may encompass.

Students responding ‘Maybe’ framed responses in terms of career choice – with some having interest in another career as well as medicine, and others feeling that it was too early to decide.

*“Not definitely sure what I want to be yet. Lot of pressure choosing.” (Written response).*

Overall, Session 1 identified a lack of clear or detailed knowledge about what a medical career may involve. The second and third sessions explored this further.

### Session 2 and 3

#### Identification of occupations

Table 4 shows that groups correctly identified most of the medical occupations as doctors, although forensic pathologist and microbiologist were identified only by a minority. However, few of the non-medical occupations were correctly identified.

**Table 4:** Frequency of correct identification of medical and non-medical occupations

Medical occupations	Session 2	Session 3	Non-medical occupations	Session 2	Session 3
General practitioner	8 (100%)	8 (100%)	Dentist	5 (62%)	4 (50%)
Paediatrician	8 (100%)	8 (100%)	Pharmacist	5 (62%)	3 (38%)
Radiologist	8 (100%)	8 (100%)	Optician	4 (50%)	3 (38%)
Anaesthetist	7 (88%)	8 (100%)	Psychologist	4 (50%)	5 (62%)
Cardiologist	7 (88%)	7 (88%)	Midwife	3 (38%)	2 (25%)
Psychiatrist	7 (88%)	6 (75%)	Chiropractor	2 (25%)	4 (50%)
Surgeon	7 (88%)	8 (100%)	Paramedic	2 (25%)	2 (25%)
Forensic pathologist	2 (25%)	2 (25%)	Physiotherapist	2 (25%)	3 (38%)
Microbiologist	2 (25%)	2 (25%)	Podiatrist	1 (12%)	1 (12%)

Frequency indicates the number of 8 groups to correctly identify each response

Some occupations were unfamiliar (podiatrist, chiropractor) and participants were largely guessing. However, more familiar occupations (paramedic, midwife) were still frequently misidentified as doctors. This reinforced the impression from Session 1 that students (and in some cases, teachers) do not have a clear idea of what jobs medical school qualifies people to do.

#### Identification of and interest in activities

Table 5 summarises the number of groups which identified each of the activities as part of a doctors’ role and the extent to which each was of interest to participants. While some of the more popular activities may be expected from their presentation in the media (eg ‘examining dead bodies’) there were also popular choices which may not be recognised as roles in medicine (eg ‘developing new treatments or drugs’). There was notable interest in empathic/communication roles, but these were poorly identified as roles performed by doctors. Many participants therefore expressed interest in roles which they do not associate with medicine, and so may not consider medicine as a career.

**Table 5:** Frequency of correct identification of activities and number of indications of interest

	No. groups correctly identifying activity ^a^			Expressions of interest ^b^		
Activity	Session 2	Session 3	Total	Session 2	Session 3	Total
Examining dead bodies from a crime scene	8 (100%)	7 (88%)	15 (94%)	30 (14.9%)	24 (12%)	54 (13.5%)
Examining dead bodies to work out the cause of death	7 (88%)	7 (88%)	14 (88%)	15 (7.5%)	16 (8%)	31 (7.7%)
Working with sports teams and athletes	1 (12%)	1 (12%)	2 (13%)	13 (6.5%)	17 (8.5%)	30 (7.5%)
Looking after children and young people when they are in hospital	6 (75%)	7 (88%)	13 (81%)	8 (4%)	21 (10.5%)	29 (7.2%)
Working in the Army/RAF/Navy	5 (62%)	7 (88%)	12 (75%)	12 (6%)	17 (8.5%)	29 (7.2%)
Talking to people with mental health problems	5 (62%)	2 (25%)	7 (44%)	11 (5.5%)	16 (8%)	27 (6.7%)
Helping people with cancer	8 (100%)	8 (100%)	16 (100%)	10 (5%)	16 (8%)	26 (6.5%)
Performing operations	8 (100%)	8 (100%)	16 (100%)	18 (9%)	7 (3.5%)	25 (6.2%)
Developing new treatments or drugs	5 (62%)	6 (75%)	11 (69%)	12 (6%)	13 (6.5%)	25 (6.2%)
Looking after babies when they are born prematurely	4 (50%)	5 (62%)	9 (56%)	12 (6%)	7 (3.5%)	19 (4.7%)
Researching new ways to try and cure diseases	5 (62%)	4 (50%)	9 (56%)	8 (4%)	7 (3.5%)	15 (3.7%)
Diagnosing illness from X-rays and scans	8 (100%)	7 (88%)	15 (94%)	8 (4%)	2 (1%)	10 (2.5%)
Talking to people about their everyday problems	1 (12%)	4 (50%)	5 (31%)	4 (2%)	6 (3%)	10 (2.5%)
Putting people to sleep before an operation	8 (100%)	8 (100%)	16 (100%)	9 (4.5%)	0	9 (2.2%)
Tracking the spread of diseases and trying to prevent spreading	7 (88%)	7 (88%)	14 (88%)	2 (1%)	6 (3%)	8 (2%)
Teaching students	2 (25%)	5 (62%)	7 (44%)	2 (1%)	5 (2.5%)	7 (1.7%)
Helping people overcome disability	2 (25%)	4 (50%)	6 (38%)	5 (2.5%)	2 (1%)	7 (1.7%)
Caring for people at the end of their life	2 (25%)	2 (25%)	4 (25%)	5 (2.5%)	2 (1%)	7 (1.7%)
Looking through a microscope to diagnose diseases	8 (100%)	8 (100%)	16 (100%)	6 (3%)	0	6 (1.5%)
Finding out what people are allergic to	8 (100%)	7 (88%)	15 (94%)	2 (1%)	3 (1.5%)	5 (1.2%)
Helping elderly people	1 (12%)	5 (62%)	6 (38%)	4 (2%)	1 (0.5%)	5 (1.2%)
Delivering babies by performing an operation (C-section)	7 (88%)	8 (100%)	15 (94%)	1 (0.5%)	3 (1.5%)	4 (1%)
Giving injections	7 (88%)	8 (100%)	15 (94%)	1 (0.5%)	3 (1.5%)	4 (1%)
Performing CPR (resuscitation) to try and save someone’s life	7 (88%)	7 (88%)	14 (88%)	1 (0.5%)	2 (1%)	3 (0.7%)
Helping pregnant women if they develop problems	2 (25%)	8 (100%)	10 (63%)	1 (0.5%)	2 (1%)	3 (0.7%)
Prescribing medicines to people	5 (62%)	6 (75%)	11 (69%)	1 (0.5%)	1 (0.5%)	2 (0.5%)
Sending people home from hospital	5 (62%)	5 (62%)	10 (63%)	0	1 (0.5%)	1 (0.2%)
Organising the delivery of healthcare in a region	3 (38%)	1 (12%)	4 (25%)	0	0	0
Developing campaigns to improve the health of everyone - stopping smoking, sexual health	0	0	0	0	0	0
Working for a company to make sure people’s workplaces are safe	0	0	0	0	0	0
Total				201	200	401

^a^The number of 8 groups per session, 16 in total, to correctly identify each response as part of doctors’ work

^b^Each participant was given two adhesive dots to allocate to the available activities to indicate which attracted them most. They could give two to the same activity, one each to separate activities, or allocate one or neither. The total is the sum of those allocated, which may be less than the number distributed to participants

Finally, we considered the changes in stated expressions of interest in medicine, along the physical ordinal scale, between the beginning and end of the session. Table 6 gives the aggregate frequencies of participants at each point of the scale before and after the sessions. Treating this as an ordinal scale, there was a clear shift in attitudes towards being more likely to consider medicine (*p* < 0.001, Mann Whitney U test, STATA Version 13.0).

**Table 6:** Combined frequencies of interest in medicine pre- and post-intervention for Session 2 and 3

	Definitely not	Probably not	Maybe	Definitely
Pre-intervention	15 (7%)	47 (23%)	103 (51%)	36 (18%)
Post-intervention	8 (4%)	25 (12%)	113 (56%)	55 (27%)

Includes data from both Session 2 (*n* = 101) and Session 3 (*n* = 100)

Interestingly, accompanying teachers occasionally commented on student categorisation of medical careers with incorrect information. From informal discussions between students and teachers during the sessions, it became evident that some teachers were lacking in basic knowledge about medical careers. For example, one incorrectly identified ‘podiatrist’ as a medical profession. Others provided incorrect basic information on selection criteria and required A-levels, course content, course duration and careers paths. Rather than directly correct teachers, researchers used this as an opportunity to open discussion around these areas in order to educate both students and teachers. This incidental finding reflects student concerns that some school staff are ill-equipped to provide accurate information.

### Summary of year 9 sessions

In the Year 9 sessions we identified knowledge gaps around the range of careers doctors can have and the types of work activities these involve. We found that an interactive session increased expressions of interest in medicine. We cannot rule out the effect of peer influence and a conformity effect in some sessions, nor whether there was an effect of expectations leading to changes in expressed views. However, groups appeared to interact naturally and we conclude that the observed effect is robust.

## Discussion

We have presented data collected from school students who were at two different points in career decision-making: those who are just beginning to think about subject choices to enable higher education applications and those who are about to apply to university.

Our findings indicate several factors which undermine young people’s awareness of medicine as a possible career and ways in which those gaps may be addressed.

Fundamentally, the challenges were rooted in knowledge gaps which may decrease or distort awareness of medicine as a possible career. Participants in these workshops emphasised a need for clear, practical information in distinct areas. These are detailed below.

### The application process

Schools that do not regularly send students to medical school may lack staff with the knowledge and time to prepare students for application. We noted incorrect information being provided by some teachers on the application process, length of the course and nature of the job. Ensuring that schoolteachers as well as students are engaged and informed may be key to reaching potential applicants.

Many Widening Participation students have limited personal contacts who have studied in Higher Education or the medical field or attend schools with limited experience of supporting medical school applications. Information sources exist on preparing an application for medicine and entry examinations such as the UKCAT, but students were not aware of these. Medical schools could clearly signpost students to this information or provide direct support through outreach.

### Understanding medicine and medical careers

For younger students, the main knowledge gap related to the nature of medicine and breadth of medical careers, meaning that students may have a limited view of what medicine involves and the range of career options available within the profession. Students appeared to recognise diagnostic and procedural activities in medicine but did not identify other activities including research, training, service development, public health or the spectrum of patient groups. This may contribute to perceptions that medicine is a narrow career.

These early perceptions of what a career in medicine involves could also have impact on the future workforce. For example, ‘talking to people with mental health problems’ was identified as a medical role by less than half of the groups, yet this skill is a major part of many specialties, including GP, emergency medicine and psychiatry – all of which have problems with recruitment and retention [21]. A lack of awareness of the range of activities common in medicine, combined with an over-emphasis on the ‘exciting’ emergency elements of medicine in some media may deter applicants who are attracted to the more empathetic aspects of medicine.

A short intervention with Year 9 students addressed some career knowledge gaps and increased their consideration of medicine but online materials and open days may reach a wider audience. Medical schools may focus on outreach events for this age group with materials to explain the scope of a career in medicine.

Older students raised concerns about the political context of the NHS and careers in medicine. Notably, research sessions took place when a dispute around junior doctors’ contracts in the UK was gaining traction in national media. Whilst we did not explore the detail around these concerns, the British Medical Association has expressed concern that doctors who move abroad following training may in future be required to repay training fees [22] and this may disproportionately deter applicants from poorer backgrounds [23]. Medical schools should recognise that students are socially and politically aware and be prepared to address these concerns.

### Work experience

Students were unclear about the role of work experience, what experience was useful and how to access it, particularly when they had no medical family members. Although medical schools recognise that work experience can be difficult to come by, experience of clinical environments may help students make better decisions and medical schools could take a more active role in this process. Medical schools should also more clearly emphasise that non-clinical experience of working with people is relevant and useful. There were also barriers in the way NHS organisations offer and arrange work experience, therefore universities may work with NHS organisations to facilitate and promote work experience options.

### Course content, university life and finance

Students were uncertain about the academic challenges of Higher Education, the change in learning style required and social issues such as accommodation and adjusting to university life. This was particularly pertinent where students had few relatives and contacts to approach. Medical schools could provide outreach sessions in association with existing university undergraduate advice services. This information may be delivered by early-stage medical students, which were a preferred source of information about the course over senior university staff.

Although students felt socioeconomic status influenced access to knowledgeable advice and support, participants did not express concerns about elitism in medical school itself. This is in contrast to earlier findings. A focus group of 14–16-year-old students found adverse stereotypes of medical students as elitist were off-putting [16], while an interview study of mature medical students from working-class backgrounds reported a predominant ‘identity conflict’ [24] due to perceived social elitism in medical schools. We found students focused more on structural than personal factors. This could have been due to methodology, which may have elicited a focus on external deterrents rather than personal feelings. Equally, the emphasis on perceived inequality of opportunity, rather than intergroup difference, may mean that cultural barriers are not as rigid as earlier authors have suggested.

Finally, the cost of medical training is a concern for students and signposting to information about student finance and NHS bursaries will be essential. Medical schools should note that NHS student bursaries are changing [25] and anticipate how these changes may affect Widening Participation students.

#### Social capital as a barrier

The knowledge gaps identified may be salient to all prospective applicants, regardless of their socioeconomic background. However, they are likely to be more acute for those without knowledgeable support at home or school.

While we did not set out to consider barriers in theoretical terms, our findings indicate the importance of knowledge from formal and informal social networks. These may be seen as a form of social capital. Social capital is a sociological concept which describes aspects of an individual’s social context, including information, which can be translated into human capital [26]. Effectively, it is a mechanism by which social power relations are replicated. It has been widely considered in the context of education [27], and found to be an influence on access to higher education among particular groups [28].

#### Modes of support

Deficits of social capital cannot necessarily be simply remediated, but recognising it is not just knowledge, but support which may be lacking may help to shape strategies.

It is not necessarily enough that information is available; it must be accessible to and navigable by the target population. This too can vary with the home and school context of the student. Widening Participation students may need additional guidance on how to find and navigate resources if they do not have people around them who can point them in the right direction. Younger school students in particular may need school teachers or careers advisors to introduce the possibility of medical career if it is not part of their home milieu.

We recommend that medical schools review how they collate and signpost information, whether provided online or face-to-face. For online resources, potential measures include search engine optimisation – ensuring that appropriate web pages are returned to naïve students’ searches (we found that one relevant blog was not returned in the first pages of Google hits). A list of ‘frequently asked questions’ prominently accessible from medical schools’ course information and admissions web pages could address students’ practical concerns.

First-hand accounts have the potential to address uncertainties about course content and careers. These could take the form of written or video blogs from existing students and junior doctors, or talks and question and answer sessions in outreach and open day events. The involvement of those from similar backgrounds was identified as particularly helpful by some participants – providing not just knowledge, but also role-modelling of people who may not fit a socioeconomic stereotype.

Finally, while open days and visits are helpful, these were also seen as time-consuming in a busy school term with exam pressures ahead. As McLachlan noted [14], outreach events may be more effective. Visits to schools may reach those who may not volunteer for open days, or be identified by teachers as being interested. If they encourage interaction with peers, they may have value in supporting those who feel isolated. Students who may particularly benefit from these initiatives include younger students, those not yet considering medicine and those who are undecided.

#### Limitations

The study has some limitations which we acknowledge here.

The study took place within a single medical school; therefore, findings may not be transferable to other locations. However, the locality is geographically and socioeconomically diverse and we have captured a range of views from many Widening Participation students.

The sample of participants was outside the control of the researchers. While there was very high participation among those attending MaD days, the risk of selection bias nonetheless exists at two levels: students are selected by their schools to attend MaD days, and school participation is itself subject to self-selection. The issues uncovered may therefore not be representative of students in other schools. Despite this, identifying problems faced by even a small proportion of the theoretical population is still potentially useful.

Methodologically, the need to fit into the existing format of the MaD days necessitated a pragmatic approach to data collection with a difficult-to-access group. This involved compromises between the time available and the depth of data accessible. Rapid interpretation of findings was needed to feed into subsequent sessions. However, the consistency of findings across different approaches gives them credibility.

The research was conducted in large part by current medical students working as student interns on the project (AM, BJB, GW and NW). While this contained a risk of bias in their having recent, and successful, experience of applications and admissions processes, their understanding was felt to be a strength in design and interpretation of the research. All sessions were observed and data reviewed by a non-clinician researcher (BB) which provided balance. No authors are currently involved in admissions processes.

## Conclusions

Students who may wish to consider medicine as a career need reliable, structured information. A lack of awareness of key areas of medical education and careers is widespread and those without access to relevant expertise at home or school have less opportunity to address this. Medical schools have a key role in widening participation and should facilitate access to reliable information through different modalities, including structured online resources and through outreach delivered by a range of staff and students.

The wide range of careers available within medicine should be emphasised in order to engage those who may not have as much interest in more high-profile areas of medicine. Medical schools may also facilitate peer support for students who feel isolated in applying to medicine and work with local NHS organisations to facilitate fair access to work experience opportunities. Future research should focus on evidence-based initiatives to inform and encourage applicants to ensure that students from less advantaged backgrounds are not excluded from medicine.

## Abbreviations

GP: General practice
MaD days: Medical and dentistry days
NHS: National Health Service
SES: Socio-economic status
UCAS: Universities and Colleges Admissions Service
UKCAT: United Kingdom Clinical Aptitude Test
WP: Widening participation
